# Can Nitazoxanide and/or other anti‐viral medications be a solution to long COVID? Case report with a brief literature review

**DOI:** 10.1002/ccr3.8162

**Published:** 2023-11-17

**Authors:** Denise D. Stewart

**Affiliations:** ^1^ Department of Life Sciences Imperial College London London UK

**Keywords:** acyclovir, anti‐viral medication, COVID‐19, herpesviruses, latent virus reactivation, long COVID, Nitazoxanide, post‐acute sequelae (PASC), SARS‐CoV‐2, shingles

## Abstract

**Key Clinical Message:**

Findings here imply lingering of virus, SARS‐CoV‐2, in the body for months. Thus, Nitazoxanide and/or other anti‐viral medications might be potential options to combat long COVID. This could transform treatment for long COVID patients globally.

**Abstract:**

Long COVID or post‐acute sequelae of COVID‐19 (PASC) continues to affect many people even after a relatively mild acute illness. Underlying causes of PASC are poorly understood. There is no particular treatment or management program developed yet. Thus, the possibility of well‐known, safe anti‐viral medications use against PASC is proposed here.

## Graphical abstract

Patient‐X Symptoms and Duration: Red lightning bolt points to the Nitazoxanide treatment time, which was the breakthrough for her recovery.

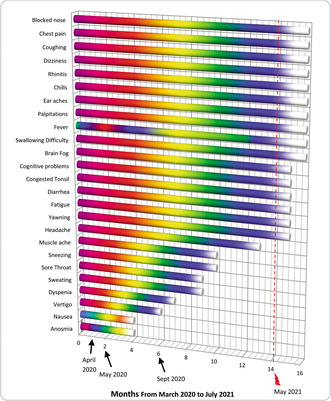

## 1 BACKGROUND

**FIGURE 1:**
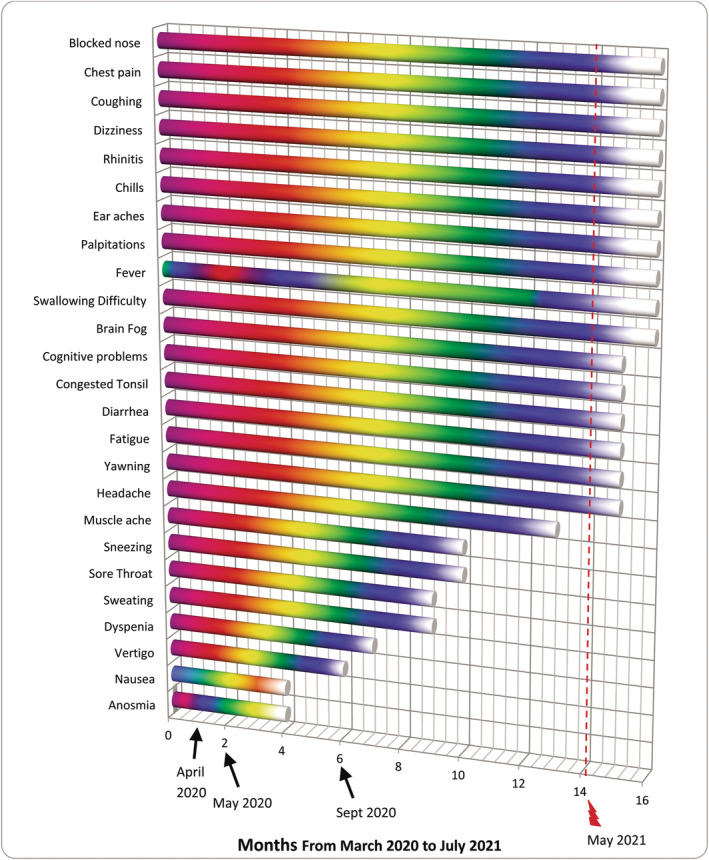
Patient‐X Symptoms and Duration: Arrows point to the times Patient‐X called doctors. Red lightning bolt points to the Nitazoxanide treatment time, in May 2021. The intensity of the symptoms was variable with periodic exasperation as explained in the main text. They are *roughly* represented with the multicolored bars comparable to a pain scoring system from 0 to 5 with: pink/red (equivalent to score 5): most intense (+++++) > blue (equivalent to score 4): ++++ > green (equivalent to score 3): +++ > orange (equivalent to score 2): ++ > yellow (equivalent to score 1): least intense (+) > white (equivalent to score 0): none (−), i.e. no symptoms. Her recovery is represented with color gradually whitening. Re: Fever: She did not have fever every day: The bar is a *rough* demonstration of “on and off” regular, persistent, variable fever she experienced with yellow representing the low grade fever ~37.8°C and least frequent, green for ~38°C, blue for ~39°C and pink/red for ~40–41°C up to ~10 days in a row, on/off, and white returning to her normal ~36.5°C. Brain fog refers to memory problems and confusion, such as numbers, days, words, etc. Cognitive problems refer to issues with practical tasks like arithmetic, driving, using kitchen appliances, etc.

Most patients recover within a few weeks after severe acute respiratory syndrome coronavirus 2 (SARS‐CoV‐2) infection and developing coronavirus disease 2019 (COVID‐19). However, varying from 10% to 87% of the patients, depending on the study, continue to be troubled by a variety of symptoms for months, including upper/lower respiratory, gastrointestinal, neurological problems and fatigue (e.g., symptoms listed in Figure 1) leading to a condition recognized as post‐acute sequelae of COVID‐19 (PASC) or long COVID.^1^, ^2^, ^3^, ^4^ Reasons for the development of PASC are not well‐understood but mainly are attributed to viral persistence in certain tissues and/or damage to the immune system leading to hyper‐inflammation particularly by autoantibodies, autoimmunity, and priming of the immune system from molecular mimicry.^1^, ^2^, ^3^, ^4^ Furthermore, neurotropic reactivation of pathogens such as herpesviruses due to COVID‐19‐related immune dysregulation; damage from the acute SARS‐CoV‐2 infection in certain organs, frequently to heart, lungs, and muscles; impacts of SARS‐CoV‐2 on microbiata, including virome; deconditioning; dysregulation of brainstem and/or vagus nerve signaling; coagulation or clotting issues via endothelial dysfunction; relapse or reinfection are also thought to be among the contributing factors.^1^, ^2^, ^3^, ^4^ Relapse, that is, worsening of symptoms, is usually associated with exertion and multiple reinfections are found to increase the chances of PASC.^1^, ^2^ These can promote new onset of cardiovascular, thrombotic, and/or cerebrovascular conditions besides Type‐2 Diabetes, myalgic encephalomyelitis/chronic fatigue syndrome (ME/CFS), dysautonomia and postural orthostatic tachycardia syndrome.^1^ Anti‐viral medications are used to treat acute COVID‐19 patients.^5^, ^6^ Nonetheless, an effective treatment for PASC is yet to be developed. An unusual PASC case with a literature review is presented below. Using an anti‐parasitic/anti‐viral medication, Nitazoxanide, with or without other medications for PASC sufferers is proposed, considering the significant recovery observed in this patient. This suggestion is made here for the first time.

## 2 CLINICAL PRESENTATION

**FIGURE 2:**
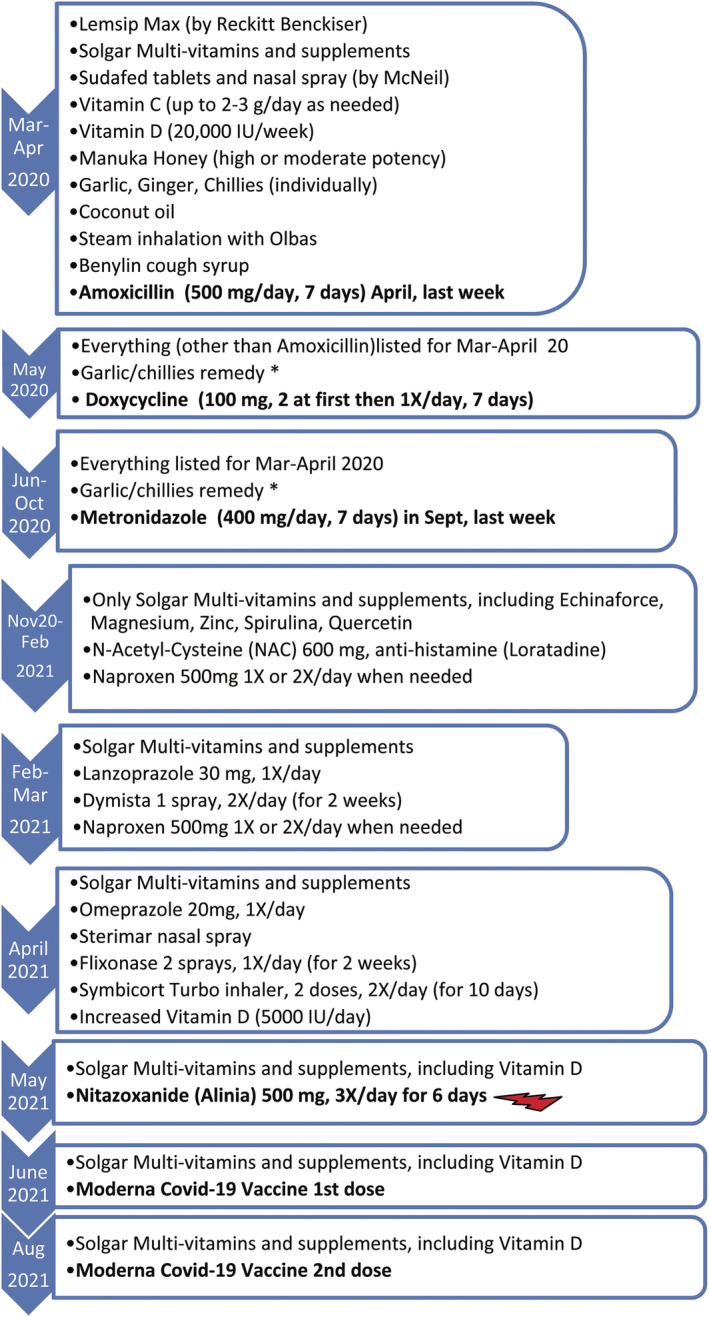
Patient‐X Treatments timeline: The over‐the‐counter medications listed were used as directed by the manufacturer when needed depending on the intensity of the symptoms. They were not all used at the same time, particularly Paracetemol containing tablets Sudafed, Lemsip or Aspirin. The supplements included probiotics by Holland & Barrett, mostly after antibiotics taken. The antibiotics and Naproxen (used around January/February 2021) were not effective for her, which also indicated that her symptoms were not related to secondary bacterial infections nor hyper‐inflammation. Nitazoxanide treatment made the biggest difference, indicated with red lightning bold. *See Appendix S1 for details of garlic/chillies remedy and Table S1 for further details. Metronidazole in September 2020 was prescribed against *Blastocystis Hominis* although she was never diagnosed with this parasite but her husband was. She had given stool sample to be analyzed for pancreatic enzymes, Calprotectin and Elastase in February 2020 during her check‐up prior to the pandemic. There was no indication of parasites nor those enzymes nor any other abnormality in her sample. Due to the pandemic, doctors were limiting testing and appointments; their GP (general physician or primary care physician) thought that potential parasite might have been lowering her immune system, prolonging her recovery time from COVID‐19 and long COVID symptoms. However, she did not benefit from Metronidazole, which was why the gastroenterologist she consulted prescribed Nitazoxanide that was the breakthrough to her recovery. She had also taken additional antibiotics due to a tooth infection and the subsequent tooth extraction at the end of May (~20 days after she had taken Nitazoxanide) however by then she had already recovered from her PASC symptoms (Figure 1).

Patient‐X, a 48‐year‐old female, had symptoms of COVID‐19 in March 2020, including sore throat, continuous dry cough, fever (38–39°C), chills, sweat, and muscle pains. Her symptoms worsened in ~2 weeks with congestion in sinuses and swollen tonsils, leading to breathing difficulties, fever up‐to 40–41°C, chills, pools of sweat, significant chest pain, lungs feeling like burning, anosmia, and diarrhea (Figure 1). She had no remarkable underlying conditions other than insulin‐resistance and slightly elevated cholesterol (Table 1). Doctors visited her at home in April 2020. They made the diagnosis of COVID‐19 and possible COVID‐19‐related pneumonia based on symptoms. She was given a course of Amoxicillin for potential secondary chest infection and took some over‐the‐counter medications (Figure 2). She called doctors again in May and September 2020 due to persistent symptoms and was given antibiotics, Doxycycline and Metronidazole, respectively. Metronidazole was given against a potential parasite, *Blastocystis Hominis*, because her husband was recently diagnosed with it and in case she might have also been infected. Owing to the pandemic, she was not tested for parasites in September 2020. Her check‐up in February 2020, prior to the pandemic, did not detect any parasites (Figure 2).

**TABLE 1:** Patient‐X Relevant Characteristics and Clinical Investigation.

Characteristics	Patient‐X				Normal range
	Pre‐COVID‐19	During COVID‐19	Post COVID‐19	Latest	
Age (Years)	48	48.5	49	51	
Sex		Female			
BMI	28	29	29	28	18.5–24.9: normal 25–29.9: overweight >30 obese
Race		White			
Duration of symptoms		~14 months			
Smoking status		Never			
Immunocompromised		No			
Hb1Ac	**46**	**47**	**46**	**47**	20–41 mmol/mol 42–47 mmol/mol: insulin resistance/pre‐diabetic
Chest X‐ray	Clear	Clear	Clear	Clear	
Sputum culture **(not checked for SARS‐CoV‐2)**		Negative for bacteria in Sept 2020			
Allergy test		Negative			
Cardiac MRI		**Minor Pericardial Effusion**			
Echocardiogram	Normal (Feb 2020)	**Minor Pericardial Effusion**	Normal No Pericardial Effusion (Sept 2021)	Normal	
Electrocardiogram (ECG)	Normal	Abnormal P wave, Q wave, Subtle ST segment change inferolaterally	Normal	Normal	
Electroencephalogram (EEG)		Normal			
Troponin			3	5	0.0–14 ng/L
Platelets	395 × 10^9^	382 × 10^9^	297 × 10^9^	369 × 10^9^	150–400 × 10^9^/L
Fibrinogen			2.9		1.6–4.8 g/L
INR (Prothrombin time)			0.9		0.8–1.1 ratio
APTT ratio			0.94		0.85–1.19 ratio
Ferritin	43	47	51	71	30–400 μg/L
ESR	18	21		16	1–23 mm/h
C‐reactive protein	3	4	4.5	3.1	0.0–10.0 mg/L
Cortisol	259				100–540 nmol/L
Cholesterol	**5.54**	**6.8**	**5.6**	4.9	3.3–5.2 mmol/L
Triglycerides	**2.92**	**3.03**	**3.19**	**2.54**	0.8–2.0 mmol/L
LDL	2.82	**4.1**	2.8	2.6	0.0–3.0 mmol/L
HDL	1.39	1.28	1.28	1.2	1.1–2.6 mmol/L
Cholesterol/HDL ratio	3.98	**5.3**	4.3	4.08	0.0–5.0 ratio
Vitamin D	59	82	68	77	50–174 nmol/L
Antibodies (against Spike protein)		259	2500		≥0.8 U/mL
Autoimmune profile				Negative/Normal	
Paraneoplastic screen				Negative/Normal	
Toxocariasis screen				Negative	
Toxoplasma screen				Negative	
HIV			Negative		

*Note*: Abnormal values are indicated in bold. If no value presented, it was not available or not applicable. Autoimmune Profile: ANA (anti‐nuclear antibody), ANCA (anti‐neutrophil cytoplasmic antibodies), ENA (extractable nuclear antigen), protein electrophoresis, rhSSeumatoid factor, anti‐CCP (anti‐cyclic citrullinated peptide), paraprotein, Bence Jones protein (for multiple myeloma), ganglioside antibodies, paranodal antibodies, serum free light chains, anti‐neural antibodies, liver and gastric periatal cell antibodies, including anti‐Gastro Periatal Cell, anti‐Smooth muscle, anti‐Mitochondrial, anti‐Liver/Kidney Microsomal, thyroid antibodies. Paraneoplastic Screen: Intracellular paraneoplastic antibodies (Hu, Yo, Ri, amphiphysin, CV2, Ma1, Ma2, Tr, ZIC4), Purkinje cells, other Cerebellar cells, IgG White matter (myelin). Toxocariasis/Toxoplasma Screen: To check on potential infections caused by pet parasites, ELISA‐Novalisa Toxocara canis IgG was used. HIV: Human Immunodeficiency Virus. Pre‐COVID‐19: Dec 2019‐Feb 2020, During COVID‐19: March‐Apr 2021, Post‐COVID‐19: Aug‐Sept 2021, after recovery time, Latest: Oct 2022‐June 2023. See Table S1 for further details.

Abbreviations: APTT, Activated Partial Thromboplastin Clotting Time; ESR, Erythrocyte Sedimentation Rate; HDL: High‐Density Lipoprotein.INR, International Normalized Ratio; LDL, Low‐Density Lipoprotein.

Antibiotics, including Metronidazole did not help and the sputum test for bacteria was negative in September 2020 (Table 1). She still experienced regular cough, blocked right nostril, swollen, and congested right tonsil besides chest pain in the upper‐right lung‐quadrant, on and off fever, chills, severity varying depending on activities. Diarrhea, bloated stomach, and cramps led her to a gastroenterologist in November 2020, who prescribed her Nitazoxanide as a precaution against the potential parasite, *Blastocystis Hominis*. She did not take Nitazoxanide immediately.

She saw ENT (ear, nose, and throat) and lung specialists in February and March 2021, respectively, due to worsening upper and lower respiratory symptoms. She was prescribed further medications (Figure 2) but she did not improve after trying them for ~2 weeks. Eventually in May 2021, she took Nitazoxanide: 1‐to help against the potential parasite (although she was not diagnosed with it—but it might have been dysregulating her immunity, thereby affecting her recovery from PASC), 2‐because of Nitazoxanide's anti‐viral properties.^5^ Having completed a course of 500 mg Nitazoxanide three times daily for 6 days, she noticed a remarkable improvement. In ~3 days, she felt the blocked nostril opened, swollen tonsil was normalized; congestion and cough were cleared shortly, after ~14 months of suffering these symptoms. The rest of her problems disappeared soon as well (Figure 1); she resumed a normal life, including exercising without relapse. She then got vaccinated in June 2021, August 2021 (for 2nd dose), December 2021 (3rd dose), and December 2022 (4th dose).

She went through reinfections in August 2021, December 2021, June 2022, September 2022, and January 2023 after traveling. These were milder as compared to pre‐May 2021 experience and she recovered faster within ~5–10 days depending on the exposure—except the reinfection in June 2022 lasting ~7 weeks. Probably, the antibodies in her system (Table 1) after COVID‐19 vaccinations helped her recovery from reinfections without going into PASC again.

## 3 DISCUSSION

These observations suggest the potential use of existing, available, safe medication, Nitazoxanide, which has anti‐viral properties, for PASC sufferers to help speed‐up recovery. There was ~1‐month gap between the patient taking Nitazoxanide and her 1st dose of vaccination. Therefore, her significant recovery was attributed to Nitazoxanide. Limitation of the study includes unavailability of testing for COVID‐19 at the beginning of the pandemic when her symptoms began in March 2020, besides sequencing to differentiate between persistence versus reinfection of the virus in 2021. Her physicians had eliminated other causes of her symptoms and thought she suffered COVID‐19 (particularly due to anosmia) and then PASC, which can be considered sufficient for diagnosis.^1^, ^7^, ^8^, ^9^ She did not have such symptoms prior to the pandemic. Her full blood counts, electrolytes, kidney, liver function, allergy, autoimmune, and paraneoplastic screen tests were within normal range before, during and post‐pandemic, ruling‐out allergies and other causes for her symptoms, including human immunodeficiency virus (HIV), toxoplasmosis, and toxocariasis (Table 1 and Table S1).

Other medications (in Figure 2) she had taken did not help her symptoms, which confirmed that her symptoms were not due to hyper‐inflammation nor any other bacterial infection—also verified by sputum test. Unfortunately, her sputum in September 2020 (Table 1) was not tested for SARS‐CoV‐2 due to test unavailability. Nevertheless, she was isolated at home mostly since her initial symptoms from March 2020 until November 2020; therefore, it is highly unlikely that she was affected by other pathogens and reinfections.

Currently, the cause of PASC is elusive.^1^, ^2^, ^3^, ^4^ Such long recovery time and persistent symptoms draw pathophysiological parallels to ME/CFS and other coronaviruses, SARS‐CoV‐1 and Middle Eastern Respiratory Syndrome coronavirus (MERS‐CoV),^1^, ^2^, ^3^, ^4^ which may be credited to large similarities in genomic sequences across them.^3^ The varying degree of intensity in her symptoms were on the same locations: mainly cough, fever, chills, chest pain in the right‐upper lung‐quadrant, blocked right nostril, congested, and swollen right tonsil. Particularly considering fever and chills, these symptoms implied persistence of the original virus, possibly multiplying while mutating as observed by others.^1^, ^10^, ^11^, ^12^, ^13^ Her fast recovery after taking Nitazoxanide also suggested that her symptoms were likely to be due to the presence of virus for months as in other studies^1^, ^10^, ^11^, ^12^, ^13^ rather than autoimmune reactions (from which she was cleared—Table 1). Some inflammation due to virus particles circulating might have additionally been contributing to her symptoms; however, she had taken anti‐inflammatory, Naproxen and N‐Acetyl‐Cysteine in January 2021 to no‐avail (Figure 2). Her blood tests, including inflammatory markers, ferritin, ESR, and C‐reactive protein (CRP) levels (Table 1, Table S1) were within normal range, indicating lack of hyper‐inflammation.

### 3.1 Initial viral load could matter to PASC

Chronic SARS‐CoV‐2 infections are frequently observed in immunocompromised individuals,^1^, ^10^, ^11^, ^12^, ^13^ yet she was not immunocompromised nor obese nor elderly people. Although there is one patient here, this is one of the rare presentations of PASC lasting for ~14 months in non‐immunocompromised patients. She could have been helped earlier if she had access to an anti‐viral medication sooner. Prior to the pandemic, she had been volunteering at a hospital, using public transport in rush‐hours besides various appointments in crowded environments. Therefore, her initial viral‐load might have been high, instigating PASC with such upper/lower respiratory symptoms and on/off fever that long. SARS‐CoV‐2 viral load is associated with increased disease severity, causing higher rate hospitalization and mortality.^14^, ^15^, ^16^ For example, the patient's husband (Patient‐Y), whose serendipitous *Blastocystis Hominis* infection led to Nitazoxanide treatment for Patient‐X, also had COVID‐19 symptoms from March 2020 onwards. His initial symptoms were similar to Patient‐X with anosmia, cough, fever, upper/lower respiratory, and gastrointestinal problems (Figure S1). Other than gastrointestinal issues, his repeat respiratory cycles over the following months were relatively milder compared to hers. This might have been partly due to his viral load being lower since he wasn't in crowded areas like his wife.

### 3.2 Genetic and/or immunological predisposition could matter to PASC

Heterogeneity across PASC patients in their predominant symptoms and multiple immunological factors is recognized.^1^, ^2^, ^3^, ^4^, ^17^ Consequently, the effected organ systems could differ in PASC patients: For instance, PASC patients showing gastrointestinal problems were discovered to contain newly expanded cytotoxic CD8^+^ and CD4^+^ T‐cell populations as well as bystander activation of Cytomegalovirus‐specific T‐cells.^17^ Furthermore, SARS‐CoV‐2‐specific CD8^+^ T cells of gastrointestinal PASC patients exhibited undifferentiated phenotypes during acute disease and elevated cytotoxic characteristics at PASC state. Conversely, in patients with respiratory‐viral symptoms, SARS‐CoV‐2‐specific T cells followed the opposite trend.^17^

Similarly here, Patient‐X was mostly affected on the sinuses, tonsils, and lungs while her husband, Patient‐Y, in the gastrointestines, particularly by nausea, diarrhea, bloated stomach, besides vertigo (Figure S1). SARS‐CoV‐2 affects gastrointestines of COVID‐19 patients regularly.^1^, ^10^, ^11^, ^17^, ^18^, ^19^ They were from the same home, potentially infected by the same virus strain in March 2020, exhibited diverging symptoms as PASC progressed. This could have been because of differences in their genetic predisposition including differential immune responses.^17^ Although low cortisol levels and increased autoantibodies were observed in PASC patients according to some studies,^1^, ^17^ this tendency did not seem applicable for these patients here (Table 1, Table S2).

In June 2022, both Patient‐X and Patient‐Y tested positive for ~3 weeks by lateral flow tests after traveling. Once more, Patient‐X had symptoms mainly in upper/lower respiratory areas while Patient‐Y gastrointestinal, besides anosmia and dysgeusia they both experienced. They recovered fully in ~7 weeks without going into PASC again. This reiterates: 1—their symptoms listed in Figure 1 and Figure S1 were attributable to COVID‐19 although they had not had a positive test at the beginning of the pandemic due to lack of testing and 2—patients' genetic/immunologic predisposition might have been responsible from their COVID‐19/PASC symptom heterogeneity as described above.^1^, ^17^

### 3.3 Testing methods and COVID‐19/PASC

Despite these symptoms since March 2020, neither of these patients tested positive for COVID‐19 by PCR nor by lateral‐flow tests in September 2020 when first tests were available to them. It is possible by the time they were tested, the virus might have been at an undetected level in their nostrils and throats, while multiplying deeper in sinuses, lungs and gastrointestines as discovered by others.^1^, ^7^, ^8^, ^9^, ^10^, ^11^, ^12^, ^13^, ^14^, ^15^, ^16^, ^17^, ^18^, ^19^ Certainly, a negative COVID‐19 test does not mean recovery.^1^, ^7^, ^8^, ^9^, ^10^, ^11^, ^12^, ^13^, ^14^, ^15^, ^16^, ^17^, ^18^, ^19^, ^20^, ^21^, ^22^, ^23^, ^24^ Other studies showed that patients who had negative COVID‐19 PCR tests were still shedding viral RNA.^1^, ^7^, ^8^, ^9^, ^10^, ^11^, ^12^, ^13^, ^14^, ^15^, ^16^, ^17^, ^18^, ^19^, ^20^, ^21^, ^22^, ^23^, ^24^ Detection of CD8^+^ T‐cell response at a study suggested live‐virus presence even after months.^12^ This highlights the importance of virus testing by alternative methods using sputum,^9^, ^12^, ^23^ bronchoalveolar‐lavage fluid,^9^, ^21^ faeces^10^, ^11^, ^18^, ^19^ and/or lung CT.^8^, ^21^, ^22^

### 3.4 Nitazoxanide, COVID‐19, and potential for PASC

Nitazoxanide, an FDA‐approved anti‐parasitic medication against *Giardia intestinalis* and *Cryptosporidium parvum*‐associated diarrhea, is a benzamide; hydrolyzed into the active form, Tizoxanide, by plasma esterases after being absorbed from the gastrointestinal tract.^25^, ^26^ It interrupts host pathways anaerobic energy metabolism by inhibiting pyruvate: ferredoxin oxidoreductase (PFOR) enzyme‐dependent electron‐transfer reactions in protozoa and anaerobic bacteria. In aerobic bacteria such as *Mycobacterium tuberculosis*, it disrupts membrane‐potential and intra‐organism PH homeostasis via uncoupling.^25^, ^26^

Its anti‐viral efficacy was discovered while treating AIDS patients in 1990s. In vitro studies confirmed that it blocks viral replication of broad range of viruses including influenza, parainfluenza, rotavirus, syncytial, hepatitis‐C, hepatitis‐B, norovirus, dengue, yellow‐fever, Japanese encephalitis, HIV, and coronaviruses, such as MERS‐CoV and SARS‐CoV‐2.^25^, ^26^, ^27^, ^28^ The mechanism of viral inhibition is attributed to Nitazoxanide's interfering with host‐regulated pathways, such as mitogen‐activated protein kinase (MAPK), nuclear‐factor kappa‐light‐chain‐enhancer of activated B‐cells (NF‐κB), type I interferon (IFN‐1), or mammalian target of rapamycin complex (mTORC1) signaling pathways, thereby slightly differing depending on virus type.^25^, ^27^ In MERS‐CoV and other coronaviruses, Nitazoxanide prohibited nucleocapsid viral protein expression in vitro.^29^ Considering the similarity between MERS and SARS‐CoV‐2, Nitazoxanide was thought to inhibit SARS‐CoV‐2, which was demonstrated using SARS‐CoV‐2/Wuhan/WIV04/2019 infected Vero E6 cells at micromolar concentrations.^30^

SARS‐CoV‐2 binds to the angiotensin‐converting enzyme 2 (ACE2) receptors, which are highly expressed on lungs, gastrointestines, and endothelial cells.^28^, ^31^ Glycosylation is one of the key processes for this binding and fusion activity, besides its role in viral spike proteins conformation, stability, shaping viral tropism.^28^ Syncytia formation is the consequent of ACE2 expressing neighboring cells fusing into multinucleated cells upon SARS‐CoV‐2 infection and subsequent display of newly synthesized fusogenic spike protein on the host cell plasma membrane.^28^ This is considered as a hallmark of advanced lung pathology in patients affected with COVID‐19 at a frequency undetected in other lung infections before.^28^ SARS‐CoV‐2 spike glycoprotein maturation at an endoglycosidase H‐sensitive stage and fusion activity were hampered by Nitazoxanide in vitro, hindering progeny virion infectivity and syncytium‐forming ability, not only for the original SARS‐CoV‐2 Wuhan‐spike but also for Alpha, Beta, Gamma and Delta‐Spike variants.^28^ These and Nitazoxanide attenuating COVID‐19 pathogenesis were confirmed by other in vitro cultures and in Syrian Hamsters.^32^

Furthermore, Nitazoxanide decreased pro‐inflammatory cytokines TNF‐α, IL‐2, IL‐4, IL‐5, IL‐6, IL‐8, and IL‐10 productions in peripheral blood mononuclear cells, resulting in reduced viral protein accumulation and suppressed IL‐6 overproduction in mice, one of the main cytokine storm mediators in respiratory viral infections.^27^ It was also suggested that airway smooth‐muscle Ca^2+^‐activated‐Cl^−^ channels (TMEM16A –Transmembrane member 16A) were blocked by Nitazoxanide, thereby bronchodilating contracted airways, aiding recovery from respiratory illnesses.^26^, ^27^, ^33^ Consequently, it is thought that Nitazoxanide could improve outcomes in patients infected with coronaviruses, including MERS‐CoV and SARS‐CoV‐2 by reducing inflammation, cytokine storm besides bronchoconstriction and other respiratory complications.^26^, ^27^, ^28^, ^29^, ^30^, ^31^, ^32^, ^33^

Various randomized, double‐blind, placebo‐controlled clinical trials have shown that Nitazoxanide is a safe medication for COVID‐19 patients.^34^, ^35^, ^36^, ^37^, ^38^, ^39^, ^40^, ^41^, ^42^, ^43^ It reduced: SARS‐CoV‐2 viral load in ~5–7 days; requirement for supplemental oxygen; relative risk of progressing to severe illness in mild or moderate COVID‐19 patients; hospitalization time; and mortality rate compared to placebo group in moderate to severe COVID‐19 patients.^34^, ^35^, ^36^, ^37^, ^38^, ^39^, ^40^, ^41^, ^42^, ^43^ Some of these trials did not demonstrate resolution of all the COVID‐19 symptoms within the time frame of the trials,^34^, ^37^, ^38^ which might have been attributable to patients' high viral load and not waiting long enough. For instance, one trial found that Nitazoxanide reduced viral load in 5 days, resulted significantly more COVID‐19 negative patients compared with the placebo group in mild cases of COVID‐19, without symptom resolution.^37^ In a further study on hospitalized patients with COVID‐19 pneumonia, the same group^39^ observed improved clinical outcomes, symptom resolution, shorter duration for oxygen supplement besides lowered levels of CRP, D‐Dimer, ferritin levels in ~7 days in the Nitazoxanide arm. Additionally, when Nitazoxanide was administered with corticosteroids, a significant reduction in ICU admissions was observed compared with placebo or corticosteroid alone groups.^39^

Another trial showed clinical improvement on the need of oxygen supplement in ~3 days in moderate cases besides SARS‐CoV‐2 RT‐PCR negativity by Day 7 subsequent to decrease in viral load.^35^ Hospital discharge time and inflammatory parameter levels, such as D‐Dimer, ultra‐sensitive‐CRP, TNF, IL‐6, IL‐8 and lymphocyte T‐cells activation markers HLA DR on CD4^+^ T‐cells, CD38 in CD4^+^ and CD8^+^ T‐cells, and CD38 and HLA‐DR on CD4^+^ T‐cells were also decreased among patients treated with Nitazoxanide compared to placebo.^35^ Similarly, by measuring clinical parameters, a group discovered the intensity of COVID‐19 was reduced if treated early with Nitazoxanide in 150 healthcare workers exhibiting COVID‐19 symptoms, resulting fewer hospitalizations.^41^

At a retrolective study, 552 COVID‐19 outpatients were administered mainly Nitazoxanide with Azithromycin (an immunomodulatory/antiviral antibiotic) and corticosteroid Prednisone.^43^ 533/552 patients recovered with majority of symptoms disappearing in ~20 days. 312/552 were severe cases, 279/552 were given additional medication from 10 days (anti‐coagulant Rivaroxaban) to 2 months (bronchodilator Formoterol and corticosteroid Budesonide) depending on symptoms.^43^ Likewise, another group found 100% success including in severe cases of COVID‐19 using Nitazoxanide/Azithromycin by personalizing treatment for patients with/without antihistamine Loratadine and/or Non‐Stereoid‐Anti‐Inflammatories depending on their symptoms.^44^

Despite the Nitro group attached to C5 position of the Nitazoxanide Thiazole ring, which might cause concerns for toxicity, Nitazoxanide is well‐known for its safety and tolerance at approved doses, including for children.^5^, ^25^, ^26^, ^27^, ^28^, ^33^ Furthermore, a study in Mexico involved pregnant COVID‐19 patients treated with Nitazoxanide effectively at 600 mg for 5 days without known harm on fetuses.^36^ Nonetheless, Tizoxanide, metabolite of Nitazoxanide, without the Nitro group, was also discovered to be as potent against SARS‐CoV‐2 and other viruses in the abovementioned studies if required.^25^, ^26^, ^27^, ^28^, ^32^, ^33^ Reducing viral replication/load and excess inflammatory response at an early stage of COVID‐19 is likely to diminish its severity, leading to symptoms resolution by supporting the innate immune system. This would reduce the risk of viral transmission besides being a preventative solution to PASC. Taken together, Nitazoxanide is likely to be effective against PASC for persisting virus as observed for Patient‐X here.

### 3.5 What about other anti‐viral medications for COVID‐19, thereby their potential for PASC?

Currently, Paxlovid, Sotrovimab, Remdesivir, and Molnupiravir are the main authorized treatment options for COVID‐19.^45^, ^46^ Paxlovid, a prodrug, combination treatment of Nirmatrelvir and Ritonavir, appears to be the most effective by 89% reduction in hospitalization or death compared to placebo for high‐risk non‐hospitalized COVID‐19 patients.^47^ Nirmatrelvir, a Proline derivative, prohibits the main SARS‐CoV‐2 protease, M^Pro^, a three domain Chymotrypsin‐like‐Cysteine protease, which processes critical viral protein precursors, thereby blocks viral replication, including for SARS‐CoV‐2 variants.^34^, ^48^ Ritonavir, an L‐Valine derivative, is an HIV‐1 protease inhibitor but it also hampers drug metabolizing protease CYP3A4 (of Cytochrome‐P450 family). It is co‐administered with Nirmatrelvir to enhance its half‐life.^34^, ^49^

Prodrug nucleoside analogues, Remdesivir and Molnupiravir, are phosphorylated to active forms in host cells, get incorporated into RNA by RNA‐dependent‐RNA‐polymerase (RdRp), causing viral replication obstruction.^34^, ^50^, ^51^, ^52^, ^53^ Remdesivir, an adenosine analogue, acts as an RNA chain terminator while the active form of Molnupiravir, beta‐D‐N4‐hydroxycytidinetriphosphate, can be directly incorporated into RNA as a substrate for cytidine and uridine triphosphates, resulting error accumulation in viral genome.^34^, ^50^, ^51^, ^52^, ^53^ Both medications reduced SARS‐CoV‐2 viral load, hospitalization or death besides improving pulmonary function for mild COVID‐19 patients.^34^, ^50^, ^51^ Their efficacy against COVID‐19 was uncertain according to some clinical trials.^52^, ^53^, ^54^, ^55^, ^56^, ^57^, ^58^ Studies continue to make Remdesivir available for oral administration^59^ but currently it is given by intravenous infusion, consequently not practical for outpatients' settings. Similarly, Sotrovimab is a neutralizing monoclonal antibody, administered intravenously and its efficacy may be reduced with SARS‐CoV‐2 variants, such as Omicron BA.2.12.1, BA.4 or BA.5.^46^, ^57^, ^60^ Nevertheless, it was found superior to Molnupiravir to reduce risk of hospital admission or death in a comparative clinical trial involving vaccinated patients infected with SARS‐CoV‐2 Omicron BA.1 or BA.2 variants, who were not hospitalized.^57^

Additionally, Acyclovir and its analogues were found effective against coronaviruses SARS‐CoV‐1, HCoV‐NK63, MERS‐CoV in vitro.^61^, ^62^ Acyclovir was successful against acute cases of COVID‐19.^63^, ^64^ It is an acyclic nucleoside analogue for guanosine and used to treat herpesvirus infections, including shingles and chicken‐pox.^65^, ^66^ Acyclovir mechanism of action in coronaviruses is unclear: It is thought to hinder: 1‐coronavirus RdRps^6^; 2‐viral gene expressions^67^; 3‐viral proteases^68^; and/or 4‐IL‐12 binding to its receptor by changing the surface, thereby reducing hyper‐inflammation.^69^

Recent studies illustrated significant correlations across COVID‐19, PASC and herpesviruses' reactivation, including Varicella‐Zoster (causing shingles), Epstein–Barr, HSV‐1 or Cytomegalovirus, attributed to COVID‐19‐induced lymphopenia.^70^, ^71^, ^72^, ^73^, ^74^ For example, at the beginning of March 2021, aforementioned Patient‐Y suffered shingles, which might have been consequential of PASC. Regardless of the cause, subsequent to completing a course of 800 mg Acyclovir 5 times daily for 7 days, he felt significantly better, all of his symptoms disappeared shortly (Figure S1), after ~12 months suffering. He resumed a normal life, including exercises. Thus, Acyclovir could be another candidate to treat PASC depending on patients' symptoms ‐if not by itself but in combination of other anti‐viral medications.^1^, ^34^, ^45^

Although no viral resistance is detected for Molnupiravir yet,^75^ resistance was observed with Remdesivir^34^, ^76^, ^77^ and Nirmatrelvir.^34^, ^78^, ^79^ Crucially, Nitazoxanide is a host‐targeted anti‐viral, therefore developing viral resistance is unlikely as compared to RdRp targeting medications such as Remdesivir, Molnupiravir when mutation rates are high on RNA viruses.^80^ Moreover, Paxlovid, Remdesivir, and Sotrovimab are not advised to use for below 12 year‐olds and Molnupiravir below 18 year‐olds nor during pregnancy unlike Nitazoxanide.^36^, ^42^, ^44^, ^46^ Paxlovid has serious drug interaction concerns for patients with comorbidities^46^ while Nitazoxanide interacts with significantly less number of drugs at moderate level, including Warfarin.^81^, ^82^, ^83^ Nitazoxanide is cheaper and more available alternative globally as compared to these COVID‐19‐approved medications.^38^, ^39^, ^40^, ^41^, ^42^, ^43^, ^44^

## 4 CONCLUSION

Besides inflammatory factors, SARS‐COV‐2 remnants lingering in the body for months in various patients leading to PASC is becoming more evident.^1^, ^15^, ^17^, ^28^, ^32^ Viral load of PASC patients is likely to be lower compared to acute infection phase of COVID‐19. Hence, Nitazoxanide's multiple moieties make it an ideal medication to treat PASC patients when the underlying cause is uncertain from virus persistence to hyperinflammation, with its potential to target the virus and inflammation as described here, if not by itself but in combination of the medications mentioned above depending on patients' symptoms. Incidentally, when administered in combination with Remdesivir, Nitazoxanide synergistically inhibits SARS‐CoV‐2 growth in vitro.^32^ This combination was successfully demonstrated in an immunocompromised, 4‐year‐old COVID‐19 patient, also demonstrating Nitazoxanide's safety for children and further potential for PASC.^84^ Systematic clinical trials with PASC patients are awaited to confirm these and this article could help pave the way.

## AUTHOR CONTRIBUTIONS

**Denise Stewart:** Conceptualization; data curation; methodology; validation; visualization; writing – original draft; writing – review and editing.

## FUNDING INFORMATION

None.

## CONFLICT OF INTEREST STATEMENT

None.

## ETHICS STATEMENT

Written informed consent for publication of their data was obtained from both patients. The prescriptions and clinical investigations were performed by their doctors upon them seeking help voluntarily due to their symptoms. This is a retrolective and descriptive review of these patients' clinical records, therefore the protocol was not submitted to an ethics committee.

## CONSENT TO PARTICIPATE

The patients' doctors had prescribed all their medications.

## CONSENT

Written informed consent was obtained from the patient to publish this report in accordance with the journal's patient consent policy.

## Supporting information


Appendix S1

## Data Availability

Yes, the raw data is available. It is already presented in the Manuscript and the Appendix S1.
