# Supplementary material for: Can Nitazoxanide and/or other anti‐viral medications be a solution to long COVID? Case report with a brief literature review
Source: Clin Case Rep. 2023 Nov 17;11(11):e8162. doi: 10.1002/ccr3.8162 (PMC10654558; doi:10.1002/ccr3.8162)
Supplement: Supplementary file 1 — Appendix S1 [file CCR3-11-e8162-s001.zip › ccr38162-sup-0001-AppendixS1.docx]

**Supplementary Information Contents:**

**Supplementary Table 1:** Blood test results for Patient-X

**Supplementary Figure 1 Legend:** Patient-Y Symptoms, Duration and Acyclovir

**Supplementary Table 2:** Clinical characteristics and Blood test results for Patient-Y

**Supplementary Method:** Clinical investigations and Medications

**Supplementary Table 1:** **Blood test results of Patient-X**

| **Blood Tests** | **Patient-X** | | | | **Normal Range** |
| --- | --- | --- | --- | --- | --- |
|  | **Pre-COVID-19** | **During COVID-19** | **Post**  **COVID-19** | **Latest** |  |
| **Haematology** |  |  |  |  |  |
| White blood cells | (7.39) 8.6*10^9^ | (7) 7.84*10^9^ | 7.6*10^9^ | (6.44) 7.9*10^9^ | (4.0-10.0) *10^9^ |
| Neutrophils | (4.8) 5.*10^9^ | (4.1) 4.7*10^9^ | 3.9*10^9^ | (3.60) 5.1*10^9^ | (2.0-7.0) *10^9^ |
| Lymphocytes | (2.0) 2.5*10^9^ | (2.2) 2.45*10^9^ | 1.7*10^9^ | (2.25) 2.3*10^9^ | (1.0-3.0) *10^9^ |
| Eosinophils | (0.07) 0.07*10^9^ | (0.1) 0.12*10^9^ | 0.0*10^9^ | (0.05) 0.05*10^9^ | (0.02-0.50) *10^9^ |
| Monocytes | (0.5) 0.5*10^9^ | (0.4) 0.51*10^9^ | 0.4*10^9^ | (0.50) 0.50*10^9^ | (0.2-1.0) *10^9^ |
| Basophils | (0.05) 0.06*10^9^ | (0.1) 0.06*10^9^ | 0.1*10^9^ | (0.04) 0.05*10^9^ | (0.02-0.10) *10^9^ |
| Platelets | (347) 395*10^9^ | (324) 382*10^9^ | 297*10^9^ | (318) 354*10^9^ | (150-410) *10^9^ |
| Red blood cells (RBC) | (5.17) 4.8*10^12^ | (4.9) 5.08*10^12^ | 4.44*10^12^ | (4.9) 4.9*10^12^ | (3.8-5.5) *10^12^ |
| Hemoglobin (Hb) | (144) 132 | (138) 139 | 129 | (138) 135 | 115-170 g/L |
| Haematocrit | (0.44) 41.5 | (0.42) 0.44 | 0.38 | (0.409) 0.420 | 0.37-0.50 L/L |
| Mean Corpuscular Vl. (MCV) | (85.5) 87.0 | (84.7) 85.6 | 84.5 | (83.3) 85.2 | 83-101 fL |
| Mean Corpusc Hb. (MCH) | (27.9) 27.7 | (27.4) 27.4 | 27.8 | (28.1) 27.4 | 27-32 pg |
| Mean Corpusc. Hb conc. (MCHC) | (326) 318 | (330) 320 | 329 | (337) 321 | 315-345 g/L |
| RBC Distribution Width | (13.7) 13.5 | (14.6) 13.2 | 14.8 | (14.2) 13.8 | 10.9-15.7 % |
| **ESR** | (21) 18 | (21) 21 |  | (19) 16 | 1-23 mm/hour |
|  |  |  |  |  |  |
| **Liver Function** |  |  |  |  |  |
| Bilirubin | (8) 6 | (7) 7 | 7 | 10 | 1.0-21.0 umol/L |
| Total Protein | (79) 74 | 79 |  |  | 60-80 g/L |
| Globulin | (31) 30 | 32 |  |  | 18-34 g/L |
| Albumin | (48) 44 | (43) 47 | 39 | 46 | 35-50 g/L |
| Alanine aminotransferase (ALT) | (21) 18 | (32) 29 | 31 | 21 | 0-55 IU/L |
| Aspartate aminotransferase (AST) | (19) 13 | 21 |  |  | 5-45 IU/L |
| Alkaline phosphatase (ALP) | (64) 58 | (74) 72 | 63 | 68 | 30-150 IU/L |
|  |  |  |  |  |  |
| **Thyroid Function** |  |  |  |  |  |
| Free Thyroxine (FT4) | (16.6) 15.6 | 15.5 |  | 19.9 | 9.0-22.0 pmol/L |
| Thyroid Stimulating H. (TSH) | (1.39) 2.3 | 1.46 |  | 1.34 | 0.35-4.94 mIU/L |
| Free T3 | (3.7) 4.0 | 3.5 |  |  | 3.1-6.8 pmol/L |
| Cortisol | 259 |  |  |  | 100-540 nmol/L |
|  |  |  |  |  |  |
| **Renal Profile** |  |  |  |  |  |
| Sodium | (140) 140 | (143) 139 | 142 | 142 | 133-146 mmol/L |
| Potassium | (4.3) 4.2 | (4.3) 4.5 | 3.9 | 4.1 | 3.5-5.3 mmol/L |
| Urea | (3.5) 3.8 | (3.9) 3.7 | 2.6 |  | 2.5-7.8 mmol/L |
| Creatinine | (53) 51 | (62) 53 | 48 | 55 | 49-104 umol/L |
| Estimated GFR | >90 | >90 | >90 | >90 | >60mL/min/1.73m^2^ |
| Uric Acid | 0.33 | 0.34 |  |  | 0.21-0.42 mmol/L |
|  |  |  |  |  |  |
| **Bone Profile** |  |  |  |  |  |
| Calcium | (2.46) 2.38 | (2.42) 2.49 | 2.29 | 2.55 | 2.20-2.60 mmol/L |
| Adjusted Calcium | 2.41 | (2.36) 2.46 | 2.32 | 2.54 | 2.20-2.60 mmol/L |
| Phosphate | 1.15 | (1.42) 1.34 | 1.10 | 1.43 | 0.8-1.5 |
| Magnesium | 0.8 |  | 0.76 | 0.75 | 0.7-1.0 |
|  |  |  |  |  |  |
| **B12 vitamin Profile** |  |  |  |  |  |
| Vitamin B12 | (398) 243 |  |  |  | 187-883 |
| Folic Acid | 13.3 |  |  |  | 2.3-17.6 |
|  |  |  |  |  |  |
| **C-Reactive protein** | (6) 3 | 4 | 4.5 | 3.1 | 0.0-10.0 mg/L |

**Supplementary Table 1 Footnotes:** Values in blue shaded brackets for **Patient-X** for *Pre-COVID-19* were from December 2019, *During COVID-19* were from November 2020 and *Latest* were from November 2022. The rest of the values for *Pre-COVID*-19 were from January 2020, *During COVID-19* were from March 2021 and *Latest* were from October 2022. *Post-COVID* values were from August 2021. If a value is not presented, it means it was not available. Her oxygen levels were 95-98% despite her symptoms since March 2020.

**Patient-X** had a check-up just before the COVID-19 pandemic and was diagnosed with insulin resistance in December 2019. The cardiac MRI in April 2021 had shown ~2 mm anterior pericardial effusion and dilated left and right ventricles (Table 1). She had developed continuous pain around her heart in August 2021. The echocardiogram performed by the emergency services in August had also shown the small pericardial effusion as well as the dilated ventricles. The subsequent check-up in Sept 2021 had shown no pericardial effusion and normal ventricles with a normal ECG (electrocardiogram) despite the presence of the pain. The stress-echocardiogram, troponin and fibrinogen measurements cleared any potential blockage in her heart and blood clots. The pain was thought to be musculoskeletal, which disappeared by December 2021. Her lung function tests had shown diminished capacity in March 2021 although chest X-Ray was normal. Due to certain neurological symptoms, she had an EEG (electroencephalogram) in November 2020 but it was normal. The symptoms were attributed to COVID-19 and long COVID by her physicians.

Her full blood counts, electrolytes, kidney, liver function tests were all within the normal range as seen in the Table above. Her **allergy tests** on skin in addition to **autoantibodies** and **paraneoplastic** screen profiles (Table 1) were negative. She was negative for HIV, COVID-19 tests PCR (Sep 2020, Dec 2020), regular lateral flow tests (Nov 2020-Dec2021) and SARS-CoV-2 Nucleocapsid protein routine antibody tests prior to vaccination in Dec 2020. Considering that her initial infection was in March 2020, by the time she was tested it might have been too late to detect COVID-19 and SARS-CoV-2 Nucleocapsid antibodies or she might have had antibodies against other SARS-CoV-2 proteins. She had antibodies against SARS-CoV-2 Spike proteins detected by Roche antibody test (259 U/ml after 1^st^ vaccine in July 2021 and 2500 U/ml after 2^nd^ vaccine in September 2021). A study [*Ni L, Ye F, Cheng M, et al. Detection of SARS-CoV-2-specific humoral and cellular immunity in COVID-19 convalescent individuals. Immunity 2020;* ***52****(6):971–7.* [*https://doi:10.1016/j.immuni.2020.04.023*](https://doi:10.1016/j.immuni.2020.04.023)] reported the presence of high titres of neutralizing anti-Spike IgG, but no antibodies were detected against the Nucleocapsid proteins in recovered COVID-19 patients, suggesting that anti-Spike IgG persists longer than anti-Nucleocapsid IgG, which might explain the results observed here. Along with the humoral immune response, Ni *et al.* also observed a Spike protein-specific T cell-population producing IFN-γ, which further contributes to conferring protective immunity against SARS-CoV-2 infection.

**Supplementary Figure 1 legend: Patient-Y Symptoms and Duration:**

**Arrows** point to the times Patient-Y contacted doctors. **Lightning bolt** points to the **anti-viral medication Acyclovir** treatment for Patient-Y treatment in **March 2021**. The intensity of the symptoms was variable with periodic exasperation. They are *roughly* represented with dark **navy blue** representing the most intense and **white** representing no symptoms: comparable to a pain scoring system from 0 to 5 with **5** being the most intense as well as more frequent (*i.e.* **darkest navy,** such as **diarrhoea graph bar,** first 4 months); **1** representing very mild besides least frequent (*i.e.* **very light blue**, such as **sneezing graph bar** at 2^nd^ month) and **0** representing no symptoms (*i.e.* **white**). **Brain fog graph bar shade** at first 3 months represents **intensity level 4**. **Headache graph bar shade** at first 3 months is equivalent to **intensity level 3**. **Vertigo graph bar shade** in June is equivalent to **intensity level 2.** His recovery is represented with colour gradually **whiten**ing. **Re: Fever:** He did not have fever every day: The bar is a rough demonstration of on/off regular, persistent, variable fever he experienced for ~a month with **dark navy** representing ~40-41 ^0^C up to ~10 days in a row on/off and **white** representing normal ~36.5 ^0^C. Shades in between represent the low grade fever ~38-39 ^0^C.

Patient-Y’s initial symptoms were similar to Patient-X with cough, fever, upper/lower respiratory problems and anosmia (Supplementary Figure 1). His repeat cycles over the following months were milder compared to hers. This might have been because his viral load was probably lower since he wasn’t in crowded areas like his wife. He was affected more on his gastrointestines, particularly by nausea, diarrhoea, bloated stomach, besides vertigo (Supplementary Figure 1). Albeit rarer, SARS-CoV-2 has been found to linger and affect gastrointestines of COVID-19 patients regularly.^1-6^

He was referred to Emergency Department due to significant vertigo and nausea in June 2020, then to a gastroenterologist in August 2020. Based on his symptoms, particularly anosmia, doctors thought he had COVID-19 and then PASC after ruling out other causes (Supplementary Table 2). His check-up in August 2020 had determined that he was infected by a parasite, *Blastocystis Hominis*. He was never diagnosed with this previously and one cannot know how long this parasite was affecting him. He was treated with Metronidazole in September 2020 against the parasite and improved significantly. Nevertheless, dizziness and some digestive problems came back shortly and were attributed to PASC after eliminating other causes of his symptoms, including food allergies, autoimmune disorders and HIV (Supplementary Figure 1, Supplementary Table 2). At the beginning of March 2021, he suffered **shingles**, which might have been consequential of PASC. Recent studies showed significant correlations across COVID-19, PASC and reactivation of herpesviruses, including Varicella-Zoster (causing shingles), Epstein-Barr, HSV-1 or Cytomegalovirus.^6-11^ This is attributed to COVID-19-induced lymphopenia with significantly reduced total lymphocytes, CD4^+^ T-cells, CD8^+^ T-cells, B cells and Natural-Killer-cells as compared to healthy controls although distress associated with COVID-19 has not been overruled for such reactivation.^6-11^

Regardless of the cause, after completing a course of 800mg **Acyclovir** 5 times daily for 7 days, Patient-Y had felt significantly better, all of his symptoms disappeared shortly (Supplementary Figure 1), after ~12 months suffering. He resumed a normal life, including exercises. He was vaccinated at the end of March and then May 2021 (2^nd^ dose), December 2021 (3^rd^ dose) and December 2022 (4^th^ dose). Timing and symptoms of his reinfections and their durations were similar to his wife’s with faster recovery –except the June 2022 reinfection lasting ~7 weeks. This also implied that *Blastocystis Hominis* was not the cause of his symptoms since March 2020 and he didn’t have such problems before March 2020 and after recovery since March 2021.

**Acyclovir** is an acyclic nucleoside analogue for guanosine and used to treat herpesvirus infections, including shingles and chicken-pox.^12,13^ It’s mono-phosphorylated to be activated by virus specific thymidine kinase that is only found in virus infected cells, which makes it safe for the rest of the body and well-tolerated. It’s phosphorylated further into tri-phosphate form by other host kinases, which then gets incorporated to viral DNA, inhibiting viral DNA-polymerase, viral DNA chain elongation and replication.^12,13^ Acyclovir analogues were found effective against coronaviruses SARS-CoV-1, HCoV-NK63, MERS-CoV *in vitro*.^14,15^ Acyclovir was successful against acute cases of COVID-19.^16,17^

Acyclovir mechanism of action in coronaviruses is unclear: It’s thought to inhibit: **1-**RNA-dependent-RNA-polymerase of coronaviruses;^18^ **2-**expression of viral genes;^19^ **3-**viral proteases;^20^ and/or **4-**IL-12 binding to its receptor by changing the surface.^21^ Certain enzymes in host cells -as in the case of other nucleoside analogues such as sofosbuvir and ganciclovir^22,23^ - and/or enzymes of coronaviruses might have been activating Acyclovir in COVID-19 patients: For example, although human Cytomegaloviruses don’t express thymidine kinase, another virally encoded protein kinase (UL97) phosphorylated Acyclovir, making it effective against Cytomegaloviruses^24^ Similarly, while Epstein-Barr viruses encode thymidine kinase, another virus encoded serine-threonine kinase was responsible to phosphorylate Acyclovir.^25^ RNA-dependent-RNA-polymerase is highly conserved across coronaviruses, specifically between SARS-CoV-1 and SARS-CoV-2 with up-to 96% protein sequence similarity. Thus, Acyclovir is another good therapeutic agent candidate for COVID-19 and PASC, considering the reactivation of Varicella-Zoster, Epstein-Barr and Cytomegaloviruses in certain patients discussed above.^6-11^

**Reference List for Supplementary Figure 1 legend:**

1. Davis HE, McCorkell L, Vogel JM and Topol EJ. Long COVID: major findings, mechanisms and recommendations. Nature Rev Microbiol 2023;**21**:133–146. <https://doi.org/10.1038/s41579-022-00846-2>
2. Gaebler C, Wang Z, Lorenzi JCC, et al. Evolution of Antibody Immunity to SARS-CoV-2. Nature 2021;**591**:639–644.
3. Morone G, Palomba A, Iosa M, et al. Incidence and Persistence of Viral Shedding in COVID-19 Post-acute Patients With Negativized Pharyngeal Swab: A Systematic Review. Front. Med. 2020;**7**:562.
4. Wu Y, Guo C, Tang L, et al. Prolonged presence of SARS-CoV-2 viral RNA in faecal samples. Lancet Gastroenterol Hepatol 2020; 5: 434-435. <https://DOI:10.1016/S2468-1253(20)30083-2>
5. Natarajan A, Zlitni S, Brooks EF, et al. Gastrointestinal symptoms and fecal shedding of SARS-CoV-2 RNA suggest prolonged gastrointestinal infection. Med 2022;**3**:371–387.
6. Su, Y, Yuan D, Chen DG, et al. Multiple early factors anticipate post-acute COVID-19 sequelae. Cell 2022;**185**:881-895.
7. Bhavsar A, Lonnet G, Wang C, et al. Increased Risk of Herpes Zoster in Adults ≥50 Years Old Diagnosed With COVID-19 in the United States. Open Forum Infect Dis. 2022;**9**(5):ofac118. <https://doi.org/10.1093/ofid/ofac118>
8. Heidary F, Madani S, Gharebaghi R, Asadi-Amoli F. Acyclovir as a Potential Add-on Therapy in COVID-19 Treatment Regimens. Pharmaceutical Sciences 2021;**27**(Suppl 1):S68-S77. <https://doi:10.34172/PS.2021.38>
9. Wang F, Nie J, Wang H, et al. Characteristics of peripheral lymphocyte subset alteration in COVID-19 pneumonia. J Infect Dis. 2020;**221**:1762–9.
10. Gold JE, Okyay RA, Licht WE, Hurley DJ. Investigation of Long COVID Prevalence and Its Relationship to Epstein-Barr Virus Reactivation. Pathogens. 2021;**10**(6):763. <https://doi:10.3390/pathogens10060763>
11. Giacobbe DR, Di Bella S, Lovecchio A, et al. Herpes Simplex Virus 1 (HSV-1) Reactivation in Critically Ill COVID-19 Patients: A Brief Narrative Review. Infect Dis Ther 2022;**11**:1779–1791. <https://doi.org/10.1007/s40121-022-00674-0>
12. Majewska A and Mlynarczyk-Bonikowska B. 40 Years after the Registration of Acyclovir: Do We Need New Anti-Herpetic Drugs? Int J Mol Sci 2022;**23**:3431. <https://doi.org/10.3390/ijms23073431>
13. Huang L, Ishii KK, Zuccola H, et al. The enzymological basis for resistance of herpesvirus DNA polymerase mutants to acyclovir: Relationship to the structure of a-like DNA polymerases Proc. Natl. Acad. Sci. USA 1999;**96:**447–452.
14. Peters HA, Jochmans D, de Wilde AH, et al. Design, synthesis and evaluation of a series of acyclic fleximer nucleoside analogues with anti-coronavirus activity. Bioorganic & Medicinal Chemistry Letters 2015;**25**:2923–2926.
15. Santos IA, Grosche VR, Bergamini FRG, Sabino-Silva R and Jardim ACG. Antivirals Against Coronaviruses: Candidate Drugs for SARS-CoV-2 Treatment? Front. Microbiol. 2020;**11**:1818. <https://doi:10.3389/fmicb.2020.01818>
16. Baker, VS. Acyclovir for SA. RS-CoV-2: An Old Drug with a New Purpose. Clin. Pract. 2021;**18(1):**1584-92.
17. Baker, VS. ACYCLOVIR for SARS-CoV-2: An Old Drug with a New Therapeutic Purpose - An Observational Study. Int J of Clin Case Reports and Reviews. 2022;**10**(2). [https://doi:10.31579/2690-4861/199](https://DOI:10.31579/2690-4861/199)
18. Abuo-Rahma GEA, Mohamed MFA, Ibrahim TS. Potential repurposed SARS-CoV-2 (COVID-19) infection drugs. RSC Adv. 2020;**10**:26895-916.
19. Li Z, Yang L. Underlying mechanisms and candidate drugs for covid-19 based on the connectivity map database. Front Genet. 2020;**11:**558557. <https://doi:10.3389/fgene.2020.558557>
20. Kumar D, Kumari K, Bahadur I, Singh P. Promising Acyclovir and its derivatives to inhibit the protease of SARS-CoV-2: Molecular Docking and Molecular Dynamics simulations. Res Sq. preprint <https://doi.org/10.21203/rs.3.rs-94864/v1>
21. Far BF, Bokov D, Widjaja G, et al. Metronidazole, acyclovir and tetrahydrobiopterin may be promising to treat COVID-19 patients, through interaction with interleukin-12. J Biomol Struct Dyn 2022;**21**:1-19.
22. Chien M, Anderson TK, Jockusch S, et al. Nucleotide analogues as inhibitors of SARS-CoV-2 polymerase, a Key Drug Target for COVID-19. J Proteome Res. 2020. <https://doi.org/10.1021/acs.jproteome.0c00392>
23. Ju J, Li X, Kumar S, et al. Nucleotide analogues as inhibitors of SARS-CoV Polymerase. Pharmacol Res Perspect. 2020;e00674. <https://doi.org/10.1002/prp2.674>
24. Talarico CL, Burnette TC, Miller WH et al. Acyclovir Is Phosphorylated by the Human Cytomegalovirus UL97 Protein. Antimicrobial Agents And Chemotherapy 1999;**8**:1941–1946.
25. Meng Q, Hagemeier SR, Fingeroth JD et al. The Epstein-Barr Virus (EBV)-Encoded Protein Kinase, EBV-PK, but Not the Thymidine Kinase (EBV-TK), Is Required for Ganciclovir and Acyclovir Inhibition of Lytic Viral Production. J Virol 2010;**84(9):**4534–4542. <https://doi:10.1128/JVI.02487-09>

**Supplementary Table 2:** **Clinical** **Characteristics and** **Blood test results of Patient-Y**

|  | **Patient-Y** | | **Normal Range** |
| --- | --- | --- | --- |
|  | **During COVID-19**  **August 2020** | **Post COVID-19**  **June 2022** |  |
|  |  |  |  |
| **Age (years)** | 49 | 51 |  |
| **Sex** | Male | |  |
| **BMI** | 22 | 22 |  |
| **Race** | White | |  |
| **Duration of Symptoms** | ~12 months | |  |
| **Smoking status** | Former, none over ~25 years | |  |
| **Immunocompromised** | No | |  |
|  |  |  |  |
| **C-Reactive protein** | <0.5 | <1 | 0.0-10.0 mg/L |
| **Ferritin** | 161 |  | 30-400 µg/L |
| **Hb1Ac** | 38 | 40 | 20-41 mmol/mol |
| **Vitamin D** | 115 |  | 30-400 µg/L |
| **Cholesterol** | **5.5** | **5.9** | 3.3-5.2 mmol/L |
| **Triglycerides** | 0.75 | 1.33 | 0.8-2.0 mmol/L |
| **LDL** | **3.4** | **3.10** | 0.0-3.0 mmol/L |
| **HDL** | 1.73 | 2.20 | 1.1-2.6 mmol/L |
| **Cholesterol/HDL ratio** | 3.2 | 2.7 | 0.0-5.0 ratio |
| **Autoimmune Profile** | Negative | Negative |  |
| **Allergy Tests** | Negative |  |  |
| **Paraneoplastic Screen** |  | Negative |  |
| **HIV** | Negative |  |  |
| **Hepatitis B** (Australia Antigen) |  | Negative |  |
| **Hepatitis C** (Ab ELISA, 3^rd^ Gen) |  | Negative |  |
|  |  |  |  |
| **Haematology** |  |  |  |
| White blood cells | 5.8*10^9^ | 7.92*10^9^ | (4.0-10.0) *10^9^ |
| Neutrophils | 3.88*10^9^ | 5.38*10^9^ | (2.0-7.0) *10^9^ |
| Lymphocytes | 1.22*10^9^ | 1.44*10^9^ | (1.0-3.0) *10^9^ |
| Eosinophils | 0.12*10^9^ | 0.21*10^9^ | (0.02-0.50) *10^9^ |
| Monocytes | 0.54*10^9^ | 0.85*10^9^ | (0.2-1.0) *10^9^ |
| Basophils | 0.07*10^9^ | 0.04*10^9^ | (0.02-0.10) *10^9^ |
| Platelets | 160*10^9^ | 171*10^9^ | (150-410) *10^9^ |
| Red blood cells (RBC) | 4.45*10^12^ | 4.74*10^12^ | (3.8-5.5) *10^12^ |
| Hemoglobin (Hb) | 144 | 147 | 115-170 g/L |
| Haematocrit | 0.410 | 0.424 | 0.37-0.50 L/L |
| Mean Corpuscular Vl. (MCV) | 92.2 | 89.5 | 83-101 fL |
| Mean Corpusc Hb. (MCH) | 32.4 | 31.0 | 27-32 pg |
| Mean Corpusc. Hb conc. (MCHC) | 352 | 347 | 315-345 g/L |
| RBC Distribution Width | 12.4 | 13.1 | 10.9-15.7 % |
| **ESR** | 3 | 2 | 1-23 mm/hour |
|  |  |  |  |
| **Liver Function** |  |  |  |
| Bilirubin | 10.3 | 9 | 1.0-21.0 umol/L |
| Total Protein | 75 | 74 | 60-80 g/L |
| Globulin | 28 | 27 | 18-34 g/L |
| Albumin | 47 | 47 | 35-50 g/L |
| Alanine aminotransferase (ALT) | 24 | 38 | 0-55 IU/L |
| Aspartate aminotransferase (AST) | 28 | 39 | 5-45 IU/L |
| Alkaline phosphatase (ALP) | 42 | 73 | 30-150 IU/L |
|  |  |  |  |
| **Thyroid Function** |  |  |  |
| Free Thyroxine (FT4) | 14.3 | 17.9 | 9.0-22.0 pmol/L |
| Thyroid Stimulating H. (TSH) | 1.31 | 1.43 | 0.35-4.94 mIU/L |
| Free T3 |  | 4.3 | 3.1-6.8 pmol/L |
| **Cortisol** | 238 |  | 100-540 nmol/L |
|  |  |  |  |
| **Renal Profile** |  |  |  |
| Sodium | 140 | 137 | 133-146 mmol/L |
| Potassium | 4.6 | 4.3 | 3.5-5.3 mmol/L |
| Urea | 4.6 | 6.7 | 2.5-7.8 mmol/L |
| **Creatinine** | 89 | 76 | 49-104 umol/L |
| Estimated GFR | >79 | >90 | >60 mL/min/1.73m^2^ |
| Uric Acid | 0.32 |  | 0.21-0.42 mmol/L |
|  |  |  |  |
| **Bone Profile** |  |  |  |
| Calcium | 2.32 |  | 2.20-2.60 mmol/L |
| Adjusted Calcium | 2.25 |  | 2.20-2.60 mmol/L |
| Phosphate | 0.8 |  | 0.8-1.5 |
| Magnesium | 0.8 |  | 0.7-1.0 |
|  |  |  |  |
| **B12 vitamin Profile** |  |  |  |
| Vitamin B12 | 478 | 445 | 187-883 |
| Folic Acid | 12.1 | 8.66 | 2.3-17.6 |

**Supplementary Table 2 Footnotes:** Values for **Patient-Y** were from August 2020 (During COVID-19) and June 2022 (Post COVID-19). If a value is not presented, it means it was not available. Abnormal values are indicated in **bold**. **LDL:** Low Density Lipoprotein, **HDL:** High Density Lipoprotein. His oxygen levels were 95-98% despite his symptoms since March 2020. See Table 1 Footnotes for Autoimmune Profile and Paraneoplastic screen antibodies.

**Patient-Y** did not have a particular check-up prior to the pandemic. Helicobacter pylori test was negative in February 2021. *Blastocystis Hominis* (in August 2020) and distal gastritis (via gastroscopy in February 2021) in addition to a small sliding hiatus hernia were discovered during his check up while he was thought to be going through long COVID. One cannot know how long these were present in him, however as explained in the Supplementary Figure 1 legend and main article text, after **Acyclovir** administration due to **shingles**, his symptoms disappeared. He did not have those symptoms prior to the pandemic. This also shows that gastritis or hiatus hernia or *Blastocystis Hominis* were not the cause of his long COVID symptoms. His full blood counts, electrolytes, kidney, liver function tests were within the normal range as seen in the Supplementary Table 2 above. His **autoantibodies**, **paraneoplastic** screen and **food allergy** profiles were negative. He was negative for HIV, COVID-19 tests PCR (Sep 2020, Dec 2020), regular lateral flow tests (Nov 2020-Dec2021) and SARS-CoV-2 Nucleocapsid protein routine antibody tests prior to vaccination (August 2020). Considering that his initial infection was in March 2020, by the time he was tested it might have been too late to detect COVID-19 and SARS-CoV-2 Nucleocapsid antibodies or he might have had antibodies against other SARS-CoV-2 proteins. A study [*Ni L, Ye F, Cheng M, et al. Detection of SARS-CoV-2-specific humoral and cellular immunity in COVID-19 convalescent individuals. Immunity 2020;****52****(6):971–7.* [*https://doi:10.1016/j.immuni.2020.04.023*](https://doi:10.1016/j.immuni.2020.04.023)] reported the presence of high titres of neutralizing anti-Spike IgG, but no antibodies were detected against the Nucleocapsid proteins in recovered COVID-19 patients, suggesting that anti-Spike IgG persists longer than anti-Nucleocapsid IgG, which might explain the results observed here. Along with the humoral immune response, Ni *et al*. also observed a Spike protein-specific T cell-population producing IFN-γ, which further contributes to conferring protective immunity against SARS-CoV-2 infection.

**Supplementary Method:**

**A:** See Figure 2, Table 1 and Supplementary Tables 1 and 2 for medications and clinical investigations. **Patient-Y** did not take any particular over-the-counter medications unlike his wife other than some Lemsip, Vitamin C, probiotics if needed between March-September 2020 and **Metronidazole** (against *Blastocystis Hominis*) in September 2020. In **March 2021** he took 800 mg **Acyclovir** 5 times daily for 7 days against Shingles, which was the breakthrough for his recovery as explained in the Supplementary Figure 1 and main text.

*Ethical aspects:* Written informed consent for publication of their data was obtained from both patients. The prescriptions and clinical investigations were performed by their doctors upon them seeking help voluntarily due to their symptoms.

**B: Ingredients and instructions of garlic/chillies remedy (Modified from:** [**https://bhavnaskitchen.com/3gs-ginger-garlic-and-green-chillies/**](https://bhavnaskitchen.com/3gs-ginger-garlic-and-green-chillies/)**):**

4 garlic bulbs, 3 lemons, 4 hot chilli peppers, 1 onion, a hand size ginger. After peeling, blend all with small amount of water. Keep the paste in a jar (one prep is enough for ~10 days). Take 1 tablespoon of the paste into a cup of hot water. Add 1 sachet of Lemsip Max and 1 teaspoon of honey and leave for 5 minutes. Then drink it hot. Repeat 3 to 4 times a day with 4 hours in between due to the Paracetemol in Lemsip Max. **Patient-X** took this 2 to 3 times/day on full stomach; at times supplementing with over-the-counter Gaviscon and Omeprazole to protect her stomach. It made a significant difference clearing the phlegm in her tonsils and sinuses helping her breathe, which probably rescued her from being hospitalized. The burning feeling in her lungs was also cleared soon after taking this remedy. However, many of her other symptoms had carried on as shown in Figure 1.
